# Clinicopathological Discordance of Biopsy Findings in Proliferative Verrucous Leukoplakia: Series of 5 Cases

**DOI:** 10.3390/dj14100627

**Published:** 2026-10-02

**Authors:** Oliver V. Waldvogel, Valerie G. A. Suter, Daniel Turek, Daniel Baumhoer, Claude Jaquiéry

**Affiliations:** 1 Department of Oral and Cranio-Maxillofacial Surgery, University Hospital Basel, Spitalstrasse 21, 4031 Basel, Switzerland; oliver.waldvogel@usb.ch; 2 Medical Additive Manufacturing Research Group (Swiss MAM), Department of Biomedical Engineering, University of Basel, 4123 Allschwil, Switzerland; 3 Department of Oral Surgery and Stomatology, School of Dental Medicine, University of Bern, Freiburgstrasse 7, 3010 Bern, Switzerland; 4 Institute of Medical Genetics and Pathology, University Hospital Basel, Schönbeinstrasse 40, 4031 Basel, Switzerland; 5 Department of Pathology, HOCH Health Ostschweiz, St. Gallen Cantonal Hospital, Rorschacher Strasse 95, 9007 St. Gallen, Switzerland; 6 Basel Research Centre for Child Health (BRCCH), Petersgraben 31, 4051 Basel, Switzerland

**Keywords:** proliferative verrucous leukoplakia, oral potentially malignant disorders, incisional biopsy, squamous cell carcinoma, verrucous squamous cell carcinoma, case series

## Abstract

**Background:** Proliferative verrucous leukoplakia (PVL) is a rare but aggressive variant of oral leukoplakia. Lesions within the spectrum of PVL are characterised by high recurrence rates and high risk of malignant progression. Their multifocal and metachronous occurrence in patients necessitates close follow-up with combined clinical and histopathological examination by sampling biopsies. However, their clinical reliability is limited due to the elusive clinical presentation of PVL, as histological features can differ within a single lesion. To date, there are no studies that have investigated the diagnostic accuracy of biopsies of PVL-associated lesions. **Case Presentation:** Out of a total of 33 reviewed patients from retrieved records, five cases with discordant biopsies were identified and were analysed in more detail based on available photographic documentation and histopathological findings. The presentation seeks to illustrate the resulting diagnostic and therapeutic challenges of inconclusive biopsies in PVL. **Discussion:** In the five cases presented, divergent interpretations of biopsies appeared to be related mainly to inconclusive or superficial biopsy sampling. An incidental finding in one case was a rare occurrence of leukoplakia on a pedicled myofascial graft with subsequent malignant transformation. **Conclusions:** Due to the characteristics of PVL-associated lesions, it is likely that the discordance rates are higher than for other oral potentially malignant disorders (OPMD). When repeated biopsies remain negative despite a strong clinical suspicion of malignancy, a full-thickness biopsy including submucosal tissue or complete excision of the lesion should be considered within an interdisciplinary setting.

## 1. Introduction

Leukoplakia is one of the most frequently encountered oral potentially malignant disorders (OPMD) of the mucosa. The purely clinical term is used for the description of an abnormal keratinization presenting as predominantly white plaques or patches after the exclusion of non-OPMD-related disorders [1,2]. Meta-analysis of longitudinal cohort studies on individuals with oral leukoplakia showed a malignant transformation rate of approximately 10% [3].

However, the exact mechanisms leading to dysplasia and squamous cell carcinoma (SCC) development are still largely unclear and unpredictable. Depending on the clinical and histological appearance, a distinction is made between homogeneous and non-homogeneous leukoplakia, the latter including a verrucous subtype. Verrucous leukoplakia is characterised by its predominantly wart-like and fissured appearance with exophytic growth. Compared to other types of oral leukoplakia, these lesions show a higher malignant transformation rate of an estimated 21% [4].

Proliferative verrucous leukoplakia (PVL; also known as proliferative multifocal leukoplakia or proliferative leukoplakia) is a rare but aggressive variant of oral leukoplakia with uncertain aetiopathogenesis. As in verrucous leukoplakia, there is no clear correlation with smoking, but there is also a correlation with other common risk factors such as alcohol consumption and HPV or EBV infection [5,6,7,8,9]. The term PVL includes a histomorphological spectrum that can range from early hyperkeratosis to dysplastic changes in various degrees to SCC. Different from conventional oral leukoplakia, PVL-associated lesions are characterised by multifocal, progressive, and non-contiguous distribution with a high tendency to recur. PVL was originally described by Hansen et al. [10] in 1985 as a spectrum disorder in which an initial homogeneous hyperorthokeratosis transformed over time into multiple exophytic lesions, resistant to any form of intervention, with rapid malignant transformation into SCC. PVL-associated lesions show the highest rate of malignant transformation among all OPMDs, at approximately 66% [11,12]. Due to the multifocality of PVL, carcinomas can arise metachronously in multiple, non-contiguous mucosal sites. In comparison to non-PVL-associated SCCs, lesions show a preference for different predilection sites such as gingiva, alveolar, and buccal mucosa [13]. There are no official or widely accepted guidelines for the clinical diagnosis, and proposals for reformulated diagnostic criteria recommended by various authors are applied instead [14,15,16]. PVL shows considerable clinical and histopathological overlap with oral lichen planus (OLP) and oral lichenoid lesions. Especially in early stages, lesions may present as a flat, homogeneous leukoplakia and can display band-like or lichenoid inflammatory infiltrate on histology, so that early lesions are not uncommonly misclassified as OLP. These similarities often delay recognition and allow retrospective diagnosis at best [17,18]. As for OPMDs in general, the examination measures for diagnosing a PVL lesion include both clinical and histopathological assessment. Presence and degree of dysplasia as well as histopathological features are used for assessing the risk of malignant transformation. Although diagnostic criteria in the current WHO classification [19] have been stated for the latter, the exact correlation between clinical findings and histopathological characteristics remains challenging.

For sample collection, the use of incisional biopsies of localised clinically suspicious areas of a lesion in question is considered standard [20,21,22,23]. However, representative sampling and thus correct histopathological assessment is complicated by the non-homogeneous and verrucous appearance of PVL. Resulting underdiagnosis caused by inaccurate/non-representative sampling may result in adverse therapeutic outcomes due to the aggressive nature and high transformation rates of PVL lesions, as compared to unexpected malignancy in other OPMDs. This paper presents five cases of PVL that have shown discordances between the initial diagnosis by incisional biopsy and the histological assessment following complete excision of the respective lesions. The series aims to illustrate difficulties of representative biopsy sampling in PVL-associated lesions with respective therapeutic consequences.

## 2. Case Series

The retrospective study was performed at the Department of Oral and Cranio-Maxillofacial Surgery at the University Hospital Basel (Switzerland), following approval of the Ethics Committee of Northwestern and Central Switzerland (EKNZ; Project-ID: Req-2022-01085). All patients with a diagnosis of PVL were retrieved from the patient archives. In the absence of universally accepted diagnostic guidelines, case identification followed the currently applied clinical criteria for PVL [11,14,15,16], based on the original description by Hansen et al. [10]. The following inclusion criteria were used: (a) patients with leukoplakia in which the biopsies confirmed simple hyperkeratosis without dysplasia and which subsequently developed an exophytic and proliferative growth in the clinical course; (b) patients with existing verrucous leukoplakia, verrucous squamous cell carcinoma (VC), or SCC based on biopsy findings with history of persistent or multifocal leukoplakia. Thirty-three records were identified and subsequently screened for inconsistent findings between the initial sampling and later excisional biopsy. Following the criteria applied by Pentenero et al. [20] and Lee et al. [21], cases were further taken into account only when

1. Double histopathological diagnosis of a single PVL-associated lesion was available:
  - Incisional biopsy (provisional diagnosis);
  - Excisional biopsy (final diagnosis).
2. Incisional and excisional specimens were examined and double-checked by the same pathologist.

A summary table of the cases presented is included in Appendix A (Table A1).

### 2.1. Case 1

**Figure 1:**
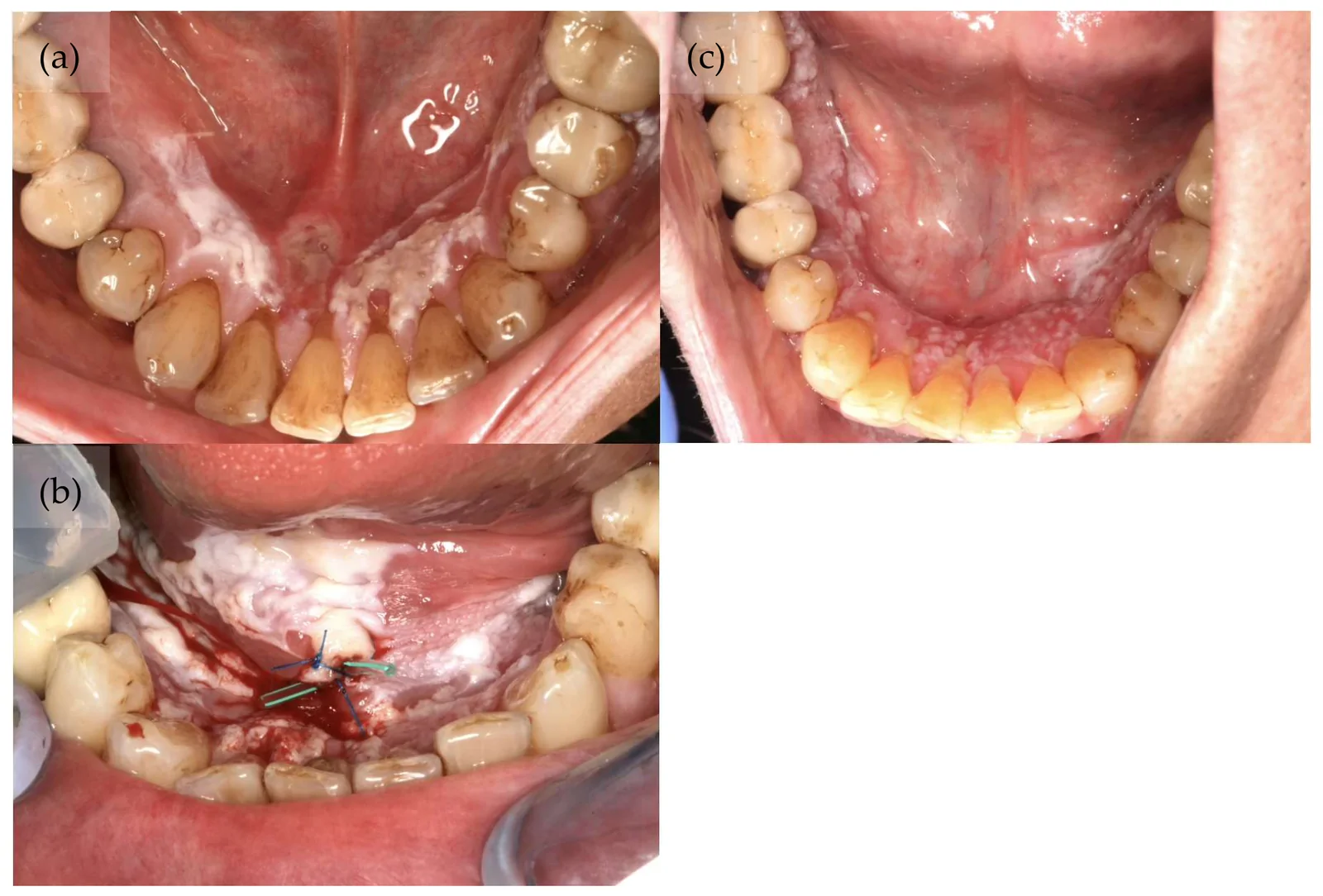
(**a**) Clinical condition as of 06/16, (**b**) presentation with duct splintage before biopsy 09/17, (**c**) expansion of the lesion as of 01/23.

A 74-year-old man was referred to our department in February 2008 due to painful oral intake and mucosal alterations affecting the buccal and lingual gingiva of the left mandible as well as the anterior floor of the mouth. An incisional biopsy revealed hyperorthokeratosis without evidence of dysplasia. As part of regular follow-up examinations, lesion progression was observed in June 2016, and incisional biopsies were taken from the buccal gingiva in the region of teeth 36/37 and the lingual gingiva in the region of teeth 41/42, showing hyperparakeratosis with acanthosis (Figure 1a). A local therapy attempt with topical tacrolimus (Protopic^®^) was initiated. Due to further increase in volume, an excision of the lesion in the anterior floor of the mouth with simultaneous submandibular duct splintage was performed in September 2017, revealing hyperparakeratosis without dysplasia (Figure 1b). Additional biopsies from the left lingual gingiva and left buccal mucosa in the same operation showed as well hyperparakeratosis without dysplasia. A laser ablation of the left lingual gingiva was additionally performed.

**Figure 2:**
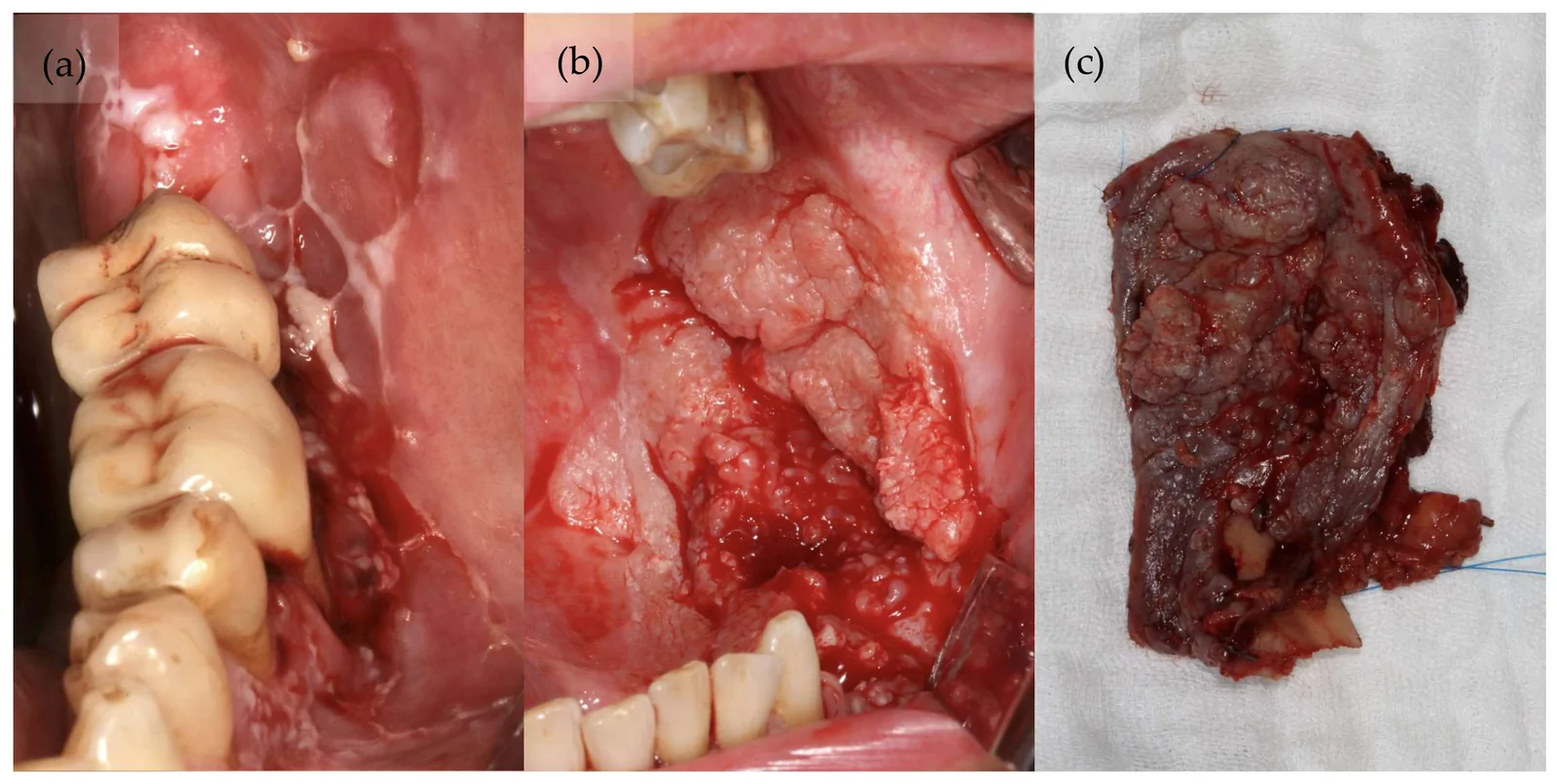
(**a**) Lesion presentation on the left buccal mucosa as of 09/17, (**b**) preoperative condition as of 08/23, (**c**) resected tumour specimen as of 08/23.

In January 2023, the patient was referred again due to progression of the lesions (Figure 1c) and the appearance of a new retromolar lesion on the left mandible with erosive features (Figure 2a). Fluconazole (Diflucan^®^) was administered to treat concomitant candidiasis. MRI and PET-CT imaging showed signal alteration with a volumetric mass in the left retromolar region, infiltrating adjacent structures. An incisional biopsy of the new lesion was performed in February 2023, which showed atypical verrucous mucosa with transition to microinvasive SCC. Further mapping biopsies performed on the lingual mucosa on the left side and the vestibular mandibular mucosa on the right side revealed atypical verrucous mucosa with high-grade dysplasia. Left retromolar tumour resection (Figure 2b,c) with microvascular reconstruction using a left radial forearm flap was subsequently performed in May 2023. Histopathological examination revealed a moderately differentiated SCC (pT3 pN0 (0/10) G2 L0 V0 Pn0 R0).

Due to the suspected malignancy of the verrucous mucosal lesion in the area of the right mandible, further resection of the right mandible was performed in August 2023 after tumour board discussion, again revealing a moderately differentiated squamous cell carcinoma (pT3 pN0 G2 L0 V0 Pn0 R0). Bilateral neck dissection (levels I–III) and microvascular reconstruction with a right radial forearm flap were conducted, followed by adjuvant radiotherapy. Subsequent vestibuloplasty with split-thickness skin grafting and implant insertion in the mandible were successfully completed. Implant placement was finalised in September 2023. Follow-up examinations have remained uneventful to date.

### 2.2. Case 2

**Figure 3:**
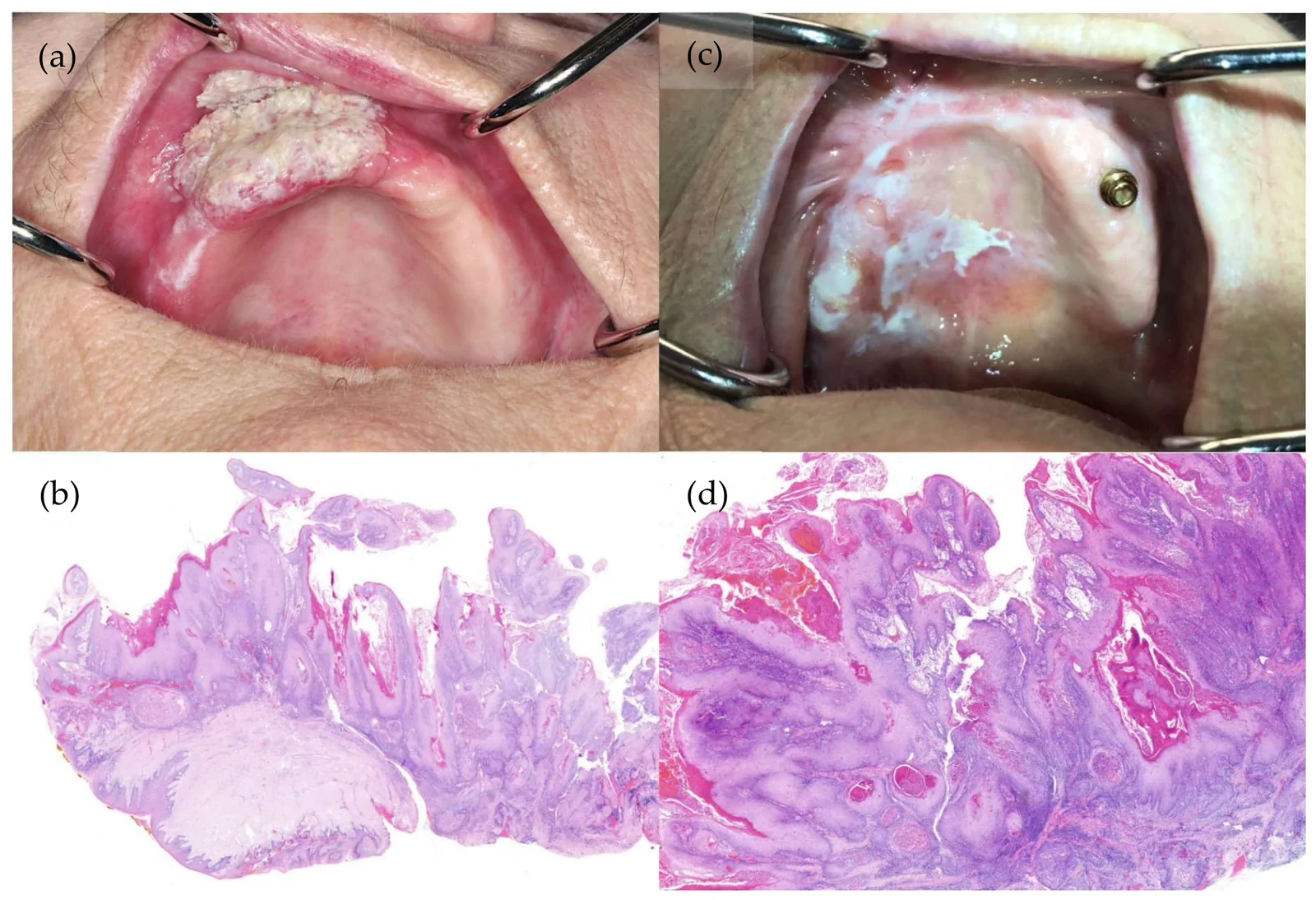
(**a**) Initial presentation as of 07/14, (**b**) histopathological specimen of complete resection showing SCC, (**c**) new lesion in the area of the flap graft in 03/16, (**d**) histopathological specimen of secondary resection showing VC.

An 80-year-old woman was referred in July 2014 with a mucosal swelling of the anterior and right maxillary alveolar ridge that had been present for about 3 months and was progressive in size. The patient reported former long-term cigarette use of an estimated 50 pack-years (py). Intraoral inspection revealed a 3.5 × 2.5 cm inhomogeneous, exophytic, verrucous lesion (Figure 3a). Histological examination of an incisional biopsy showed hyperparakeratosis with low-grade dysplasia and lymphocytic inflammation. Further grading of epithelial dysplasia was not possible due to inflammatory overlay. The lesion was completely removed in the same month on the basis of a decision by the tumour board, as the clinical presentation was suspicious for malignancy. The histopathological evaluation confirmed a moderately differentiated keratinizing SCC (Figure 3b). While resection margins were tumour-free, tumour cell nests were found in close proximity to the deep margin, so that after further discussion in the tumour board, the joint decision was made to perform a resection of the anterior wall of the maxillary sinus and the neighbouring edentulous alveolar process as well as a neck dissection level I–III on the right side. Examination of the resection specimen revealed no further tumour manifestations and tumour-free lymph nodes. The mucosa of the anterior wall of the maxillary sinus showed focal squamous metaplasia and granulocytic inflammation. Due to the resulting tissue defect with an extensive oral-antral connection on the right side caused by the previous operations, defect coverage by means of a temporalis myofascial flap was performed in October 2014. Within 2 months, the flap was fully covered by original palatal mucosa.

During follow-up in March 2016, a new leukoplakic lesion was detected in the area of the transplanted flap (Figure 3c). Incisional biopsy was performed in the transition zone to the flap, which showed verrucous leukoplakia with hyperplasia and parakeratosis. High clinical suspicion of recurrence led to complete excision of the lesion, and indeed, histologic work-up revealed VC (Figure 3d). Subsequent adjuvant radiotherapy had to be interrupted because of decreased general condition. The patient died a few months later.

### 2.3. Case 3

**Figure 4:**
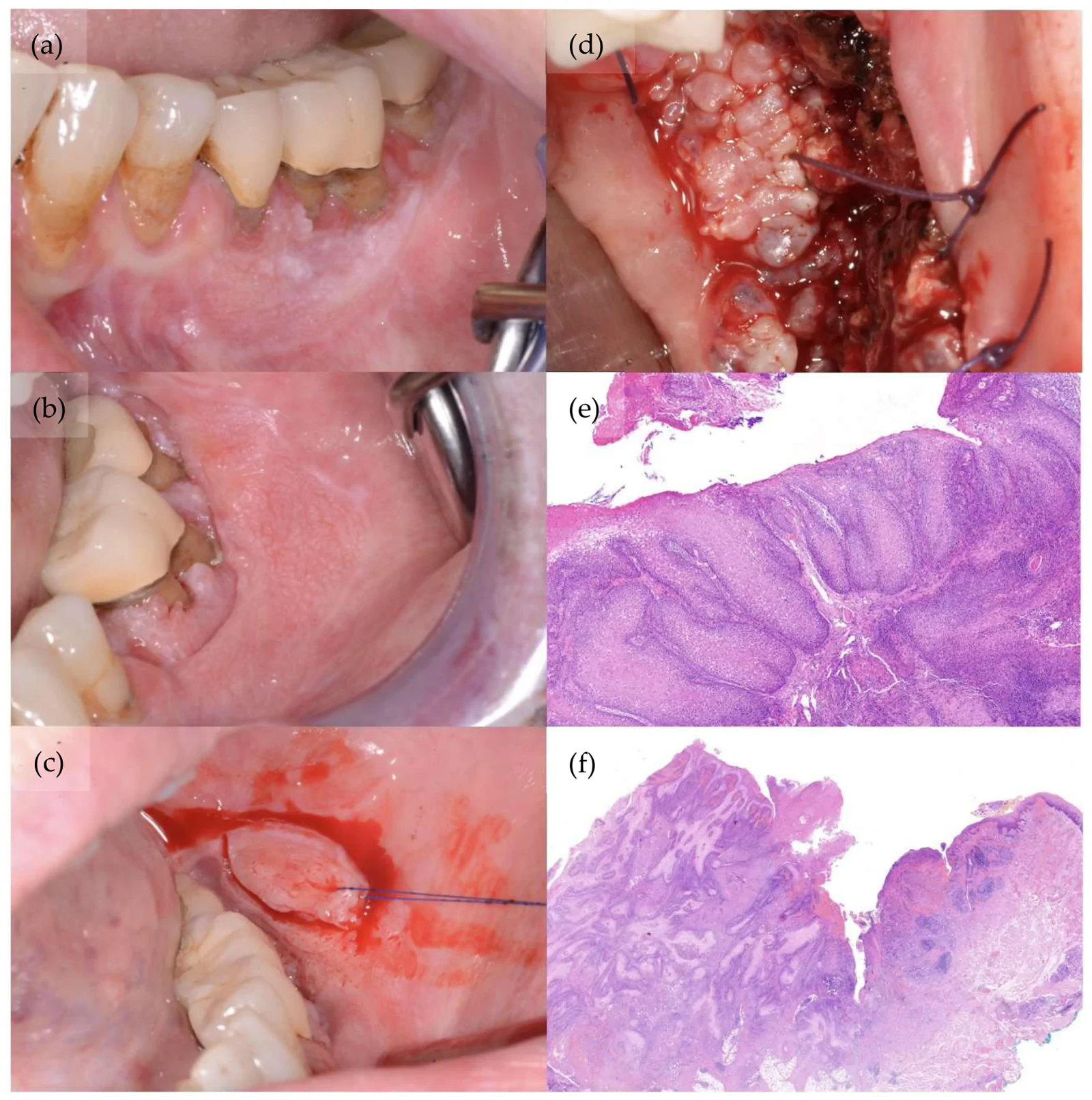
(**a**) Clinical presentation as of 11/15, (**b**) Lesion progression to buccal mucosa with speckled inhomogenous plaque in 05/17, (**c**) Incisional biopsy of the left alveolar mucosa in 05/17, (**d**) Intraoperative situs during tumour resection (06/25) in the region of the left mandibular ascending ramus, (**e**) Histopathological specimen of incisional biopsy (05/25) showing SCC (**f**) Histopathological specimen of complete resection (06/25) showing moderately differentiated SCC with transmural infiltration.

A 58-year-old woman was initially referred to the Department of Oral Surgery and Stomatology at the School of Dental Medicine of the University of Bern in March 2005. The patient denied any deleterious oral habits. Initial presentation showed multifocal mucosal alterations on the buccal mandibular alveolar ridge on the left side. Histopathological examination of the collected biopsy samples revealed homogeneous leukoplakia without dysplasia. During regular oral medicine follow-up, a vestibular and early buccal extension of the findings was detected with a new inhomogeneous plaque-like appearance in February 2014. A representative biopsy of a 1.4 cm lesion on the buccal gingiva was performed and showed chronic lichenoid mucositis without dysplasia. The clinical and histopathological diagnosis was oral lichen planus with localised proliferative verrucous leukoplakia in the area of the left alveolar mucosa (Figure 4a).

In May 2017, an inhomogeneous plaque-like lesion with speckled appearance was observed in the buccal mucosa, and the adjacent gingiva appeared thickened and irregular (Figure 4b). Incisional biopsies of the buccal mucosa (Figure 4c) and the gingiva in region 34 were taken. The pathologist reported lichenoid inflammation and noted that the features were not typical of verrucous leukoplakia and did not match PVL. However, the clinicians suspected PVL. Unfortunately, the patient did not return for follow-up examinations for several years and was referred to our department by a private practitioner in May 2025. The patient also reported new pain during food intake and difficulties with mouth opening. At the time of the initial consultation, pronounced mucosal lesions were visible on the left cheek with a palpable central induration, indicating transmural spread. In the same month, mapping biopsies were performed in the area of the left buccal mucosa, confirming the suspected diagnosis of moderately differentiated SCC (Figure 4e).

In June 2025, tumour resection was performed of the left cheek and buccal mucosa (Figure 4d), left alveolar process resection, and left neck dissection level I–V with simultaneous reconstruction using an anterolateral thigh flap (ALT flap) from the left and an extended upper arm flap from the left with facial nerve reconstruction. Histopathological examination of the resected specimens revealed a moderately differentiated, partially keratinising SCC (pT4a, pN0 (0/25), L0, V0, Pn0, G2, R0), as shown in Figure 4f. Postoperatively, adjuvant radiotherapy was performed. The patient showed an uneventful healing process and has remained recurrence-free to date.

### 2.4. Case 4

**Figure 5:**
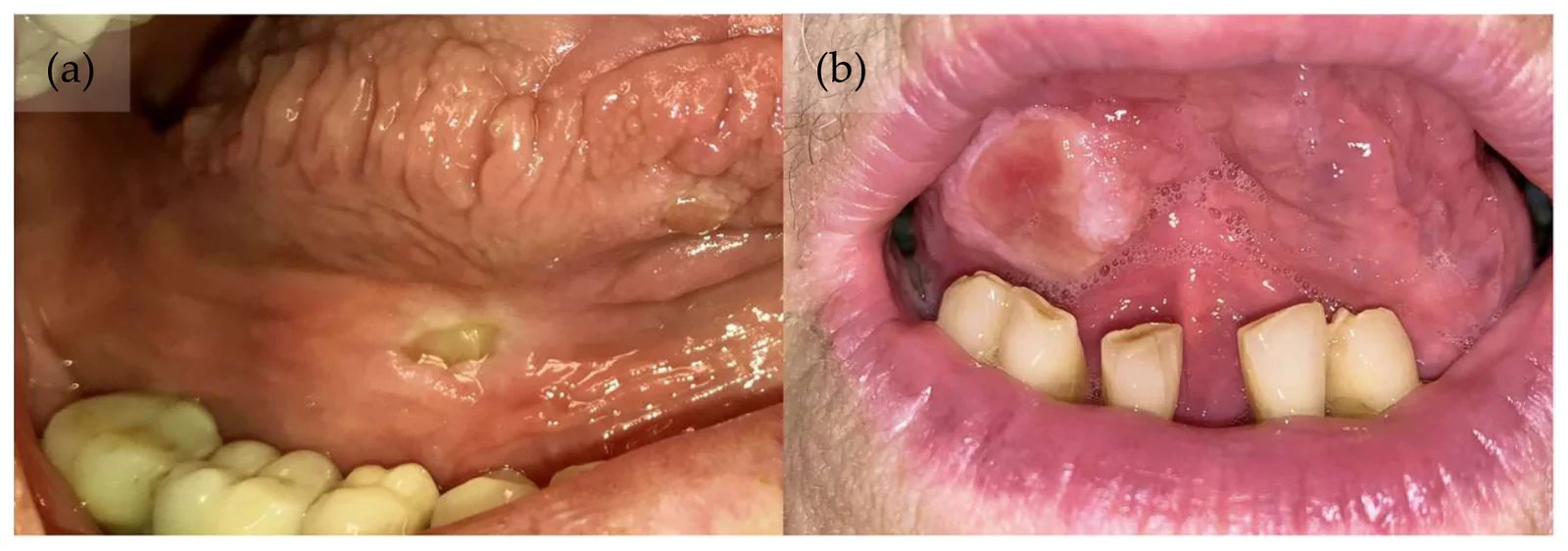
(**a**) Initial presentation in May 2021, (**b**) new lesion in September 2021.

An 83-year-old woman presented in May 2021 with a mass in the floor of the mouth with transition to the ventral tongue surface (Figure 5a). Three and a half years earlier, a mucosal lesion had been excised in the same region, which showed parakeratosis with acanthosis and nonspecific ulcerations. Intraoral examination of the current lesion showed a 2 × 3 cm measuring, indurated, wall-shaped mucosal change. Histological examination of the incisional biopsy revealed a verrucous proliferation and chronic inflammation without higher-grade dysplasia. Due to the fragmentation of the specimen and tangential incision, malignancy could not be excluded. In consequence, the lesion was completely excised 3 weeks later. Histological work-up showed a well-differentiated SCC. During a follow-up in September 2021, a new mucosal lesion was found further anteriorly on the ventral tongue surface (Figure 5b). Histology of the performed incisional biopsy showed no dysplasia or atypia. Due to a suspected recurrence, the lesion was completely removed 2 months later in accordance with the tumour board decision. Histologic examination of the specimen revealed VC. No other recurrence appeared during follow-up.

### 2.5. Case 5

**Figure 6:**
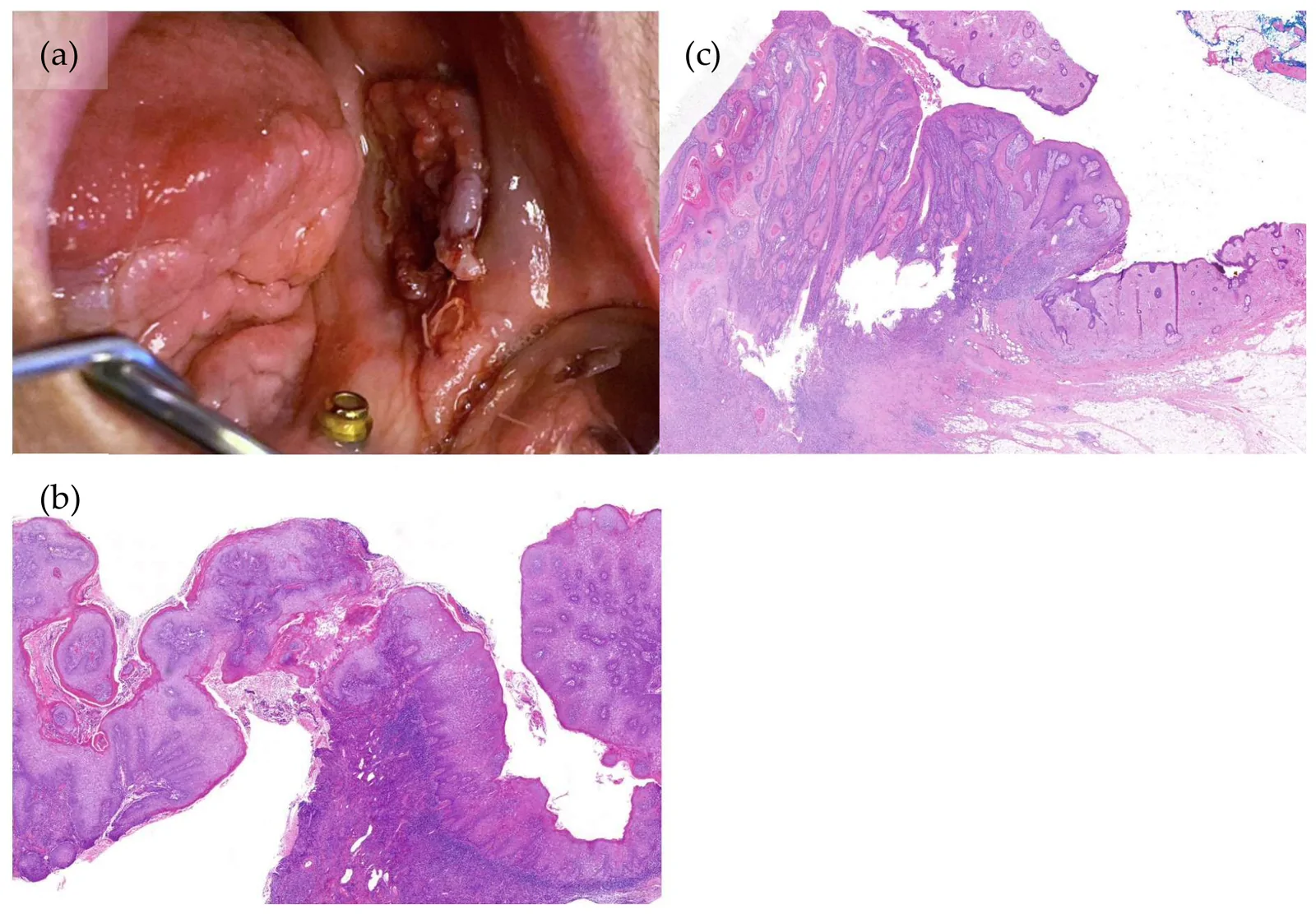
(**a**) Condition after initial incisional biopsy in the anterior region of the lesion, (**b**) histological specimen of incisional biopsy showing no dysplasia, (**c**) histological specimen of complete resection showing partly keratinized SCC.

An 85-year-old woman presented at our department in February 2020 with an inhomogeneous, partly exophytic, mucosal lesion on the left cheek, which had persisted for 3 weeks. At that time, the patient was undergoing regular clinical follow-up due to pre-existing mucosal lesions on the right floor of the mouth and the left lingual mucosa. In the past, the patient had been diagnosed with microinvasive SCC in the area of the right buccal mucosa in 2011 and in the left mandibular vestibule in 2019. The patient did not report any deleterious oral habits. Intra-oral examination revealed a 2 × 2 cm erosive-ulcerative lesion on the left lateral aspect of the anterior tongue with tenderness to palpation. An incisional biopsy was performed (Figure 6a), in which histological examination showed partially papillomatous, parakeratotic keratinizing squamous epithelium with evidence of *C. albicans* and chronic inflammation. However, histologically, there was no unequivocal evidence of cytologic dysplasia (Figure 6b). As the clinical presentation combined with rapid progression in size was suspicious for malignancy, further diagnostic work-up was performed. MRI imaging showed bone infiltration and involvement of lymph nodes. The patient presented shortly thereafter with an extraoral ulceration on the left cheek, corresponding to the intraoral lesion. A complete excision was performed 1 month after initial biopsy with subsequent continuity resection, neck dissection, and microsurgical reconstruction. Pathological examination revealed a moderately differentiated, partly keratinizing SCC (Figure 6c). The patient subsequently underwent adjuvant radiotherapy and received palliative immunochemotherapy after a new onset of VC of the tongue and the left mandibular alveolar ridge. The patient died one year later.

## 3. Discussion

The five analysed cases were treated at the Department of Oral and Cranio-Maxillofacial Surgery at the University Hospital Basel (Switzerland) between April 2011 and October 2025. Since then, two patients have died of the disease, and three remain disease-free during regular clinical follow-up. Three cases experienced multiple sampling errors during treatment. All lesions of the reported cases were biopsied using cold scalpel excision. It is notable that in all cases described, only single-site biopsies were performed. Selection of a conclusive or particularly suspicious site for biopsy sampling within a lesion was based on the subjective assessment and experience of the treating surgeon. Comparison with the histopathological findings suggested that discordant biopsy results may have been attributable to bioptic sampling at inconclusive/non-representative sites or massive inflammatory overlay. Across all five cases, the incisional biopsy underestimated the final diagnosis established on the excisional specimen. The provisional diagnoses ranged from hyperkeratosis without dysplasia and under-graded low-grade dysplasia to lichenoid inflammation not matching PVL, whereas the corresponding excisional diagnoses comprised SCC and VC. In every case, the definitive diagnosis was therefore reached only after complete removal of the lesion, which directly determined the subsequent oncological management. Overall, all specimens showed a markedly acanthotically thickened stratified squamous epithelium with pronounced hyperkeratosis and hyperparakeratosis, partly with keratin plug formation (case 2). The tissue surfaces displayed a verrucous pattern, while deeper tissue layers revealed broadening and blunting of the rete ridges, accompanied by a prominent lymphoplasmacytic inflammatory infiltrate at the epithelial–stromal interface.

The most challenging aspect in the diagnosis and treatment of PVL lesions in the five cases described seems to be the marked discordance between clinical and histopathological features. Several factors may contribute to this discordance. Apart from the location of the biopsy, the depth of the obtained tissue may be of particular relevance in PVL. As suggested by the present cases, the mucosal surface may show only a bland verrucous or hyperkeratotic pattern, whereas the features relevant to the assessment of dysplasia and early malignancy—including broadening and blunting of the rete ridges and the architecture of the epithelial–stromal interface—reside in deeper tissue layers. Because a reliable histopathological evaluation of PVL-associated lesions can rarely be based on cellular morphology alone, a specimen encompassing the full thickness of the mucosa together with the underlying submucosal tissue may allow the lesion to be assessed more completely. However, it should be emphasised that an insufficient sampling depth cannot be established as the causative factor in the present cases, since the retrospective design does not allow the effects of biopsy depth, sampling site, intralesional heterogeneity, and disease progression during the interval between incisional biopsy and lesion excision to be clearly separated. While the selection of the biopsy site remained guided by clinical experience and directed at the clinically most suspicious areas, such as indurated, ulcerated, or exophytic verrucous portions, a standardised biopsy protocol that consistently includes both mucosal and submucosal tissue could help to reduce the risk of non-representative, superficial sampling.

Close interdisciplinary collaboration and communication usually allow identification of discrepancies between the clinical and histological impression. Adverse therapeutic consequences of sampling errors could thus be partially mitigated. The decision for complete removal of a suspicious lesion despite “negative” biopsy findings was made in each case on the basis of critical clinical and/or radiological discussion in an interdisciplinary tumour board certified by the German Cancer Society (DKG). In comparison with the remaining PVL patients and the respective total number of biopsies treated at our department, the observed discordant biopsy results referred to a relatively small number of patients. Considering the entire population of treated PVL patients at the department, underdiagnosed lesions seem to cluster in certain individuals. This could be related to the interindividual differences in the dynamics and characteristics of PVL. Patients with a more frequent, metachronous occurrence of lesions, such as in PVL, should have more sampling biopsies taken and be subjected to a stricter follow-up.

As of today, there are no studies that have investigated the diagnostic accuracy of biopsies of PVL-associated lesions. The ambiguous clinical presentation and the absence of standardised nomenclature suggest that biopsy accuracy is still inferior to that of other OPMDs. For OPMDs and oral malignant lesions, there is little evidence addressing the diagnostic accuracy of biopsy procedures [20,21,22,23,24,25]. In all cited studies, rates of agreement (concordance) regarding diagnosis and degree of dysplasia between biopsy and excision of a corresponding lesion were assessed for this purpose. Surveyed concordance rates thereby ranged between 17 and 89% (Table 1). Chen et al. [23] additionally investigated reasons for discordant incisional biopsies, with sampling errors being identified as the most common cause at 60% and 23% due to histopathological discrepancies.

In this context, interobserver variability seems to be particularly high in non-homogeneous and verrucous lesions. Onofre et al. [26] showed discrepancy rates between clinical and histological diagnosis of 25% for homogeneous and 50% for non-homogeneous leukoplakia. These results were confirmed in a larger cohort study by Mutalik et al. [24], where discrepancy rates, or percentage of misdiagnosis, accounted for 21% for homogeneous and 47% for verrucous leukoplakia, respectively. Especially for non-homogeneous appearances as in PVL, histological features can differ within the same lesion, and bioptic sampling may not always be representative [20,25]. As single-site biopsies may be a sufficient and justified diagnostic method in common OPMDs, findings of single-site biopsies in PVL lesions thus seem to be problematic in terms of validity. Rates of underdiagnosis are also more frequent in non-homogeneous leukoplakic lesions (34%) than in homogeneous leukoplakia (21%), as shown by Lee et al. [21].

Therefore, the use of multiple-site biopsy examination has been recommended in several papers aiming to reduce rates of underdiagnosis and unexpected malignancy [21,22,23,27,28,29]. Multiple-site biopsies with adequate sampling depth should be weighed against diagnostic excision (complete excision) and considered on an individual basis. Especially in the case of non-homogeneous or extensive mucosal changes, diagnostic excision is associated with increased morbidity or cannot be performed at all without immediate soft and/or hard tissue reconstruction [20]. However, the comparison of available studies dealing with the sensitivity of incisional biopsies remains challenging. Inclusion criteria are often vague, and the average time between biopsy and complete excision differs considerably in some cases. However, the latter seems to be important because malignant transformation can occur more quickly, especially in non-homogeneous lesions like PVL. In this respect, studies with longer delay times might report falsely high discrepancy rates. Due to the pathognomonic occurrence of lesions in PVL, multiple collections of biopsies over extended periods of time are necessary.

The multifocal and metachronous character of PVL lesions, as well as their high recurrence rate, can be explained by the concept of oral field cancerisation, where a mucosal area of variable size may show multiple epithelial areas with genetic alterations at the same time due to the lateral clonal spread (as applicable for PVL) or simultaneous exposure to cancer risk factors [30]. Field cancerisation here seems to be of particular interest regarding case 2, where a leukoplakic lesion reappeared in a grafted myofascial flap after tumour resection. To date, there are no reports regarding the development of leukoplakic lesions in intraoral soft tissue flaps and only a few articles regarding the occurrence of SCC [31,32,33,34]. Additionally, the aetiopathogenesis of such secondary carcinomas remains mostly unclear. Physiological tissue alteration of intraoral flaps is assumed to be caused by exposure to environmental factors of the oral cavity as well as irradiation from radiotherapy.

The resulting reactive and inflammatory changes lead to a pseudomembranous appearance of the grafted flap due to epidermal hypertrophy and parakeratosis [35,36,37]. There are different approaches to explain mechanisms in the development of carcinoma in situ and SCC of soft tissue flaps. One possibility is the conventional exposure to carcinogens, which, in the case of soft tissue grafts, can proceed both before and after transplantation [38]. Gradual lateral clonal spread (i.e., creeping replacement) as originally described by Braakhuis et al. [39] is discussed as another possible reason. Closely related to the concept of field cancerisation, infiltration of the local soft tissue flap would occur by surrounding peripheral mucosal epithelium, allowing dysplastic cells to expand [38]. However, these mechanisms were proposed for skin-bearing flaps in which a transplanted epidermis persists on the flap surface. In case 2, the pedicled temporalis myofascial flap carried no skin paddle and was completely resurfaced by adjacent host oral mucosa within two months, so that the leukoplakic lesion and the subsequent VC developed in oral mucosal epithelium of host origin rather than in transplanted tissue. This does not represent creeping replacement in the strict sense, as no resident epithelium was present that could have been replaced. The covering epithelium originated entirely from the previously affected mucosal field, which is also consistent with the central location of the lesion. Ultimately, the exact aetiology of the leukoplakia on the temporalis myofascial flap remains unclear.

However, the early onset, coupled with the rapid progression and malignant transformation within less than six months, seems to be highly uncommon. Retrospectively, irritation due to the prosthetic restoration cannot be completely ruled out, despite the close clinical follow-up. Regarding this concept, Thomson and Hamadah [29] suggested the principle of so-called ‘field-mapping biopsies’ for patients presenting with multifocal lesions. To facilitate decision-making for representative biopsy specimens in lesions affecting multiple widespread areas and to simplify follow-up surveillance, clinically recognisable lesions are documented in a standardised protocol with detailed description of appearance, distribution, and anatomical location.

Several adjunctive strategies have been proposed specifically to improve the selection of representative biopsy sites in multifocal or heterogeneous lesions. Beyond the outlined ‘field-mapping’ approach, diagnostic tools such as toluidine blue vital staining and optical imaging techniques including tissue autofluorescence and chemiluminescence have been suggested to highlight clinically suspicious areas and thereby guide sampling. These methods exploit the fact that pathologically altered tissue loses its epithelial fluorophores and consequently shows reduced or absent autofluorescence. Their routine clinical use is currently not recommended owing to low-quality evidence and a considerable false-positive rate. Nonetheless, they may provide complementary information for site selection, particularly when a representative biopsy is otherwise difficult to obtain [29,40,41].

The inconsistent terminology and the lack of specific diagnostic criteria in the context of histopathological examination of PVL lesions seem to pose considerable challenges in clinical practice and research. Upadhyaya et al. [42] demonstrated a poor inter-observer agreement among pathologists assessing PVL lesions. Liu et al. [4] even suggested that epithelial dysplasia may not be an indicator for risk assessment of malignant transformation in verrucous leukoplakia at all, as many morphologic signs associated with malignant transformation seem to be absent in verrucous lesions. The current WHO Classification of Head and Neck Tumours [19] includes new uniform criteria for the assessment of dysplasia and thus makes diagnosis possible even when only architectural changes are found. This enables a more standardised histopathological classification and better comparability.

Besides the original PVL criteria according to Hansen et al. [10] based on clinical presentation and histopathological findings, patient studies often apply more recently proposed criteria [14,15,16], which are only partially in agreement. Reflecting this need for standardisation, the AAOMP/NASHNP expert consensus guideline by Thompson et al. [13] introduced a simplified descriptive terminology comprising four categories for staging the histologic findings of PVL-associated lesions: ‘Corrugated ortho (para) hyperkeratotic lesion, not reactive’ (HkNR); ‘Bulky hyperkeratotic epithelial proliferation, not reactive’; ‘Squamous cell carcinoma (SCC)’; ‘No category applicable’. The category of ‘SCC’ also includes cancer suspicion and special types like SCC with papillary architecture or the less common verrucous squamous cell carcinoma (VC), which features a lower rate of malignant transformation than the conventional type. Importantly, this terminology was explicitly developed to facilitate clinicopathological correlation rather than to establish the diagnosis on histopathology alone: the consensus emphasises that PVL represents a clinicopathological phenomenon whose histologic categories must always be interpreted in conjunction with the clinical presentation. It further underlines that the histologic spectrum is heterogeneous, that not all PVL-associated lesions display a verrucous architecture, and that a definitive diagnosis cannot rely on microscopic examination alone.

Due to the inclusion of cancer suspicion, cases that fail to fulfil definitive histologic criteria for carcinoma or display sampling artefacts can be considered likewise for a diagnosis of PVL. Each lesion can therefore be classified and evaluated for its potential for malignant transformation. In addition to more uniform histopathological diagnostics, standardised criteria for diagnosing PVL clinically have also been mentioned by the WHO Collaborating Centre for Oral Cancer/Precancer [11] and the aforementioned WHO Classification of Head and Neck Tumours [19]. Therefore, the standardised use and application of these criteria in everyday clinical practice and in research is highly recommended [9]. It is highly desirable that this will represent a substantial move towards better comparability of clinical trials with PVL cohorts, as future multicentric investigations with larger study populations are needed to enhance procedures in clinical and histopathological diagnostics of PVL.

In the context of a comprehensive follow-up, the described performance of multiple-site biopsies and precise, standardised documentation of PVL lesions should be completed by systematic photo documentation to ensure early detection and assessment of new morphological changes. Given the partially limited informative value of histopathological diagnostics in PVL, the overall clinical presentation of each patient should always be critically assessed in the synopsis of all available clinical, histological, and radiological findings over a longer period of time.

In the identified and presented cases, consistent photo documentation could not always be obtained from the records. The authors acknowledge this limitation of the present paper. Further limitations include the small number of only five patients selected from a limited institutional cohort, which precludes any estimation of representative discordance rates and limits generalizability. As with any referral-based tertiary-centre series, a selection bias towards more advanced or diagnostically challenging lesions cannot be excluded. The primary aim of this report is thus not to provide new quantitative evidence or a novel diagnostic algorithm, but rather to raise awareness of an underrecognised and clinically consequential pitfall. In addition, no standardised objective criteria were available to define representative versus non-representative biopsy sites, so the retrospective attribution of discordance to sampling remains, in part, interpretative. Finally, the observation that underdiagnosed lesions appeared to cluster in individual patients is hypothesis-generating only and must be interpreted with caution given the small number of cases; the underlying biological or clinical determinants of such interindividual variability remain to be established in larger, prospective cohorts.

## 4. Conclusions

Obtaining representative biopsies remains challenging in PVL-associated lesions. Multiple-site field-mapping biopsies of sufficient depth, including both mucosal and submucosal tissue, may help reduce the risk of sampling errors. A close interdisciplinary exchange, detailed clinical documentation, and close follow-up should be standard procedures for affected patients to rapidly detect progression and changes in suspicious lesions. Depending on the dynamics of the disease, the indication for biopsy, and consequently often for complete removal of a lesion, should be made earlier for PVL patients. Interdisciplinary cooperation and communication, in particular with the involved pathologist, are of great importance in order to limit the negative therapeutic consequences of inconclusive biopsies.

## Figures and Tables

**Table 1:** Surveyed concordance rates for oral leukoplakia.

Total Cases	Agreement, Single-Site	Agreement, Multiple-Site	ATB [mos]	Type of Lesion	Author
46	71.7%	n.a.	10.4	Non-homogeneous OPMDs	Pentenero et al. [20]
242	56%	71.4%	<1	Oral leukoplakia	Lee et al. [21]
101	49%	n.a.	4.4	OPMDs	Holmstrup et al. [22]
272	89%	83.3%	2.5	OPMDs	Chen et al. [23]
115	76.5%	n.a.	n.a.	Oral leukoplakia	Mutalik et al. [24]
23	17%	n.a.	n.a.	Non-homogeneous OPMDs	Richards et al. [25]

ATB: Average time between biopsy and complete lesion removal.

## Data Availability

Due to the nature of the research and ethical considerations, data is available upon request from the corresponding author..

## Appendix A

**Table A1:** Clinical, histopathological, and follow-up characteristics of the five presented cases of proliferative verrucous leukoplakia with biopsy discordance.

Characteristic	Case 1	Case 2	Case 3	Case 4	Case 5
Age/Sex	74/M	80/F	58/F	83/F	85/F
Risk factors	None	Former smoker (~50 py)	None	None	None
Affected sites & disease course	Buccal/lingual gingiva and floor of mouth (L mandible); later left retromolar region and bilateral mandible. Multifocal, metachronous.	Anterior/right maxillary alveolar ridge; new lesion on temporalis myofascial flap graft (field cancerisation).	Buccal mandibular ridge, buccal mucosa and gingiva (L); multifocal, with OLP/lichenoid pattern.	Floor of mouth extending to ventral tongue; earlier lesion in same region; new metachronous tongue lesion.	Left cheek; pre-existing right floor of mouth and left lingual mucosa; prior microinvasive SCC (R buccal 2011; L mandibular vestibule 2019). Multifocal, metachronous.
Follow-up	~2008–2025 (17 years)	~2014–2016 (~2 years)	~2005–2025 (20 years)	~2018–2021 (4 years)	~2011–2021 (10 years)
Provisional diagnosis (incisional biopsy)	Repeated hyper-ortho/parakeratosis without dysplasia (2008–2017); atypical verrucous mucosa progressing to microinvasive SCC (2023).	Hyperparakeratosis with low-grade dysplasia (inflammatory overlay); flap: verrucous leukoplakia.	Homogeneous leukoplakia (2005); chronic lichenoid mucositis (2014); lichenoid inflammation, not matching PVL (2017).	Verrucous proliferation, no higher-grade dysplasia (fragmented/tangential specimen; malignancy not excludable); new lesion: no dysplasia/atypia.	Papillomatous parakeratotic epithelium, chronic inflammation; no dysplasia.
Final diagnosis (excisional biopsy)	Moderately differentiated SCC, bilateral (pT3 pN0 G2 R0).	Keratinising SCC; flap lesion: VC.	Moderately differentiated, partly keratinising SCC (pT4a pN0 G2 R0).	Well-differentiated SCC; new lesion: VC.	Moderately differentiated, partly keratinising SCC.
Discordance pattern	Multiple non-dysplastic incisional biopsies over years despite progression to SCC.	Incisional under-grading (LGD → SCC); flap leukoplakia → VC.	Repeated lichenoid/non-dysplastic reports despite clinical PVL suspicion, progressing to SCC.	Incisional no/under-grading, progressing to SCC and VC.	Incisional no dysplasia, progressing to SCC.
Outcome	Alive, no recurrence	Died	Alive, no recurrence	Alive, no recurrence	Died

F: female; L: left; LGD: low-grade dysplasia; M: male; OLP: oral lichen planus; PVL: proliferative verrucous leukoplakia; py: pack-years; R: right; SCC: squamous cell carcinoma; VC: verrucous carcinoma. Tumour staging according to the TNM/UICC classification.
